# Rethinking Epithelial Ovarian Cancer Surveillance Protocols

**DOI:** 10.7759/cureus.103569

**Published:** 2026-02-13

**Authors:** Divya P Vuppu, Priya Bhati, Pranidhashree C A, Sheejamol V S

**Affiliations:** 1 Gynaecological Oncology, Amrita Institute of Medical Sciences and Research, Kochi, IND; 2 Biostatistics, Amrita Institute of Medical Sciences and Research, Kochi, IND

**Keywords:** financial burden, ovarian cancer, recurrence, surveillance, tumour marker

## Abstract

Background: This study aims to identify the main method of recurrence detection, site of recurrence, and three- and five-year disease-free survival (DFS) rates of ovarian cancer while considering the financial burden on the patient and family.

Methods: Two hundred and ten consecutive epithelial ovarian cancer cases from the hospital-based cancer registry between January 2015 and June 2023, in which patients had documented remission after completing primary treatment and were diagnosed with first recurrence, were included in the study. These four modes of detection of recurrence were considered: symptoms (abdominal pain, bloating, abdominal distention, etc.), physical findings, raised tumor marker (CA-125), and imaging.

Results: This study found that raised CA-125 is the primary detection method in 59% of cases, followed by symptoms (21.4%). Imaging identifies recurrence first in 15.2% of cases and physical examination in 4.3%. Observed patterns suggest an association between the site of recurrence - particularly the pelvis and abdominopelvic peritoneum - and the method by which recurrence was first detected. Physical exams are most effective for pelvic recurrences (72.8%), while symptoms and CA-125 levels are higher for abdominopelvic peritoneal recurrences (72.7% and 68.8%, respectively). Patients have a median of six hospital visits, travel a median distance of 70 km for each visit, incur direct costs of ₹9000, and have a median DFS of 18 months.

Conclusion: The study highlights elevated CA-125 and symptoms as primary recurrence indicators, questioning physical exam efficacy, and stresses the financial and emotional burden of frequent hospital visits.

## Introduction

Ovarian cancer has the highest mortality among gynecological malignancies, with a five-year survival rate of only 46% in developed countries [1]. Most patients experience recurrence after primary treatment, with estimated recurrence rates of 80% in advanced-stage disease and 25% in early-stage disease [2].

The Global Cancer Observatory (GLOBOCAN) 2020 highlighted significant differences in the current and projected burden of ovarian cancer between countries with high and low Human Development Index (HDI). The report predicts a substantial rise in new cases and deaths in low HDI countries, by approximately 96% and 100%, respectively, compared to a 19% increase in new cases and a 28% increase in deaths in very high HDI countries [3].

Many survivors undergo asymptomatic surveillance to detect recurrence early and potentially improve overall survival. Current surveillance guidelines for epithelial ovarian cancer are largely based on expert consensus due to limited robust data. Established guidelines, including those from the National Comprehensive Cancer Network (NCCN), the European Society of Gynaecological Oncology (ESGO), and the International Federation of Gynaecology and Obstetrics (FIGO), recommend follow-up every three months for the first two years, every six months for the next three years, and annually thereafter. Each visit typically includes a detailed history, clinical examination, measurement of tumor markers (most commonly CA-125 and carcinoembryonic antigen when relevant), and, at the clinician's discretion, annual imaging [4].

Frequent surveillance impacts patients and their families, requiring time and financial resources and contributing to anxiety and fear of recurrence. A reassessment of current surveillance protocols is necessary to develop a more patient-centered and resource-conscious approach.

The primary objective of this retrospective study was to identify the predominant mode of detecting the first recurrence of epithelial ovarian cancer. Secondary objectives included evaluating the most common site of recurrence and its relationship with the mode of detection, estimating the direct financial burden of surveillance using travel distance and consultation costs at each outpatient visit, and assessing three- and five-year disease-free survival (DFS) rates.

## Materials and methods

Ethical approval

After obtaining approval from the Institutional Ethics Committee, a single-institution retrospective study was conducted in the Department of Gynaecological Oncology at the Amrita Institute of Medical Sciences, Kochi, India (IEC-AIMS-2024 GYNEC ONCO-402).

Inclusion and exclusion criteria

All epithelial ovarian cancer patients who had CA-125 < 35 U/mL, and contrast-enhanced CT of the abdomen and pelvis with no residual disease three months after completing primary treatment, and who subsequently developed a first recurrence, were included in the study. Patients with biochemical recurrence were also included in the study. Biochemical recurrence was defined as fulfilment of the CA-125 criteria in the absence of radiological evidence of disease. Patients with no documented recurrence in our database, incomplete data, disease progression on primary treatment, or non-epithelial ovarian cancer were excluded.

Surveillance protocol

The surveillance protocol followed in our institution for all ovarian cancer patients included a visit every three months for the first two years and then every six months for the next three years. Routine contrast-enhanced CT of the abdomen and pelvis was performed annually. Additional CT scans were obtained immediately if patients exhibited clinical symptoms, abnormal examination findings, or an elevated CA-125, defined as either a value >35 U/mL, a doubling from the previous nadir, or a rise on two consecutive occasions. Each visit included a detailed history, examination, pelvic examination, and assessment of tumour markers by a gynaecological oncologist. All data were stored as electronic medical records (EMR).

Data collection

An anonymised list of ovarian cancer cases was obtained from the Cancer Registry of the Amrita Institute of Medical Sciences, Kochi, India. The study included patients with a prior diagnosis of ovarian cancer who developed disease recurrence between January 2015 and June 2023. Clinical, pathological, treatment, CA-125, and imaging data from diagnosis to first recurrence were retrieved from EMR. Deaths were ascertained from the EMR or the cancer registry.

Disease remission was defined as normal CA-125 values and no evidence of disease on imaging after completion of first-line treatment. Recurrence was considered based on the physician's diagnosis, which led to initiation of treatment.

The four modes of detection of recurrence were symptoms, physical findings, raised tumour marker (CA-125), and imaging. The primary mode of detection was defined as the method that first led to further investigations or the start of treatment. If two modes were present simultaneously, symptoms or physical findings were considered the first mode of recurrence. If elevated CA-125 led to imaging showing recurrence, CA-125 was considered the first mode. Imaging was considered the first mode in cases where recurrence was detected during annual or routine screening. Simultaneous ordering and staggered completion of investigations may lead to misclassification; however, predefined operational definitions were applied to minimise this risk.

Recurrence outcomes collected included parameters present at recurrence diagnosis, site of recurrence, time to recurrence, number of hospital visits, distance travelled to the hospital for each visit, total surveillance time, time to start second-line treatment, and last follow-up date. Sites of recurrence were grouped into five categories: pelvis, retroperitoneal and pelvic lymph node, abdominopelvic peritoneum, upper abdominal lymph node and diaphragm, and distant metastasis.

DFS was defined as the duration between diagnosis and recurrence.

Statistical analysis

The minimum sample size was calculated based on a previously reported proportion of recurrences detected by tumour markers (31%) [5]. Assuming a 95% confidence level and relative precision of 20%, the sample size was estimated using the formula:

$n = (Z&sup2; &times; p &times; (1 &minus; p)) / d&sup2;$

where Z = 1.96 for a 95% confidence level, p = 0.31, and d = 0.062. Substituting these values,

$n = (1.96&sup2; &times; 0.31 &times; 0.69) / (0.062&sup2;) &asymp; 210$

Thus, the minimum required sample size was 210 participants.

Data were analysed using Statistical Product and Service Solutions (SPSS, version 20; IBM SPSS Statistics for Windows, Armonk, NY). The most common mode of detecting the first recurrence was estimated as a percentage with a 95% confidence interval. Time between first detection and start of treatment was estimated as median and mean ± SD. Pearson's chi-square test was applied to find the association between mode of detection and recurrence. Kaplan-Meier survival analysis was used to study the overall survival and DFS. A p-value of <0.05 was considered statistically significant.

## Results

Study population

Two hundred and ten consecutive epithelial ovarian cancer cases from the cancer registry between January 2015 and June 2023, satisfying the inclusion criteria, were included in the study.

Basic demographic data

The median age of the study population was 53 years (27-79), and the median BMI was 25 kg/m² (14-45).

The majority of cases were stage III (141, 67.1%), followed by stage IV (36, 17.3%), stage I (19, 9%), and stage II (14, 6.6%). The most common histology was high-grade serous carcinoma (179, 85.2%), followed by mucinous (9, 4.3%), clear cell and endometrioid (8, 3.9%) each, low-grade serous carcinoma (4, 1.9%), and others (2, 0.8%) (Table 1). The mean duration from completion of treatment to recurrence was 12.7 months.

**Table 1:** Basic demographics and histology BMI: Body Mass Index, HGSC: High-Grade Serous Carcinoma, LGSC: Low-Grade Serous Carcinoma, SCC: Squamous Cell Carcinoma χ² and p value calculated using Pearson's chi-square test

Parameters	Total patients, n=210	Symptoms, n= 45	Physical findings, n= 9	Raised CA-125, n=124	Imaging, n=32	P value	χ² value
Age (Years)							
< 60	156 (74.2%)	35 (22.4%)	6 (3.8%)	89 (57%)	26 (16.8%)	0.61	1.73
>= 60	54 (25.8%)	10 (18.5%)	3 (5.5%)	35 (65%)	6 (11%)		
BMI (kg/m^2^)							
<23	60	10	3	35	12	0.18	4.78
>= 23	150	35	6	89	20		
Stage							
I	19 (9%)	7 (36.8%)	0	8 (42.1%)	4 (21.1%)	0.24	11.46
II	14 (6.6%)	2 (14.2%)	2 (14.2%)	6 (42.8%)	4 (28.8%)		
III	141 (67.1%)	30 (21.2%)	5 (3.5%)	87 (61.7%)	19 (13.6%)		
IV	36 (17.3%)	6 (16.6%)	2 (5.5%)	23 (63.8%)	5 (14.1%)		
Histology							
HGSC	179 (85.2%)	35 (19.5%)	7 (3.9%)	114 (63.6%)	23 (13%)	0.62	21.34
LGSC	4 (1.9%)	1 (25%)	-	2 (50%)	1 (25%)		
Clear cell	8 (3.9%)	3 (37.5%)	-	3 (37.5%)	2 (25%)		
Mucinous	9 (4.3%)	2 (22.2%)	1 (11.2%)	3 (33.3%)	3 (33.3%)		
Endometrioid	8 (3.9%)	2 (25%)	1 (12.5%)	2 (25%)	3 (37.5%)		
SCC	1 (0.4%)	-	-	-	1 (100%)		
Clear + mucinous	1 (0.4%)	-	-	-	1 (100%)		
Surveillance Time (months)		18 (12-25)	23 (10.5-36)	17 (12-27)	21.5 (15-34.25)	0.245	4.16

Mode of detection of first recurrence

The most common method of detecting recurrence was elevated CA-125 (124, 59%), followed by symptoms (45, 21.4%). Imaging was the first mode of detection in 32 (15.2%) cases and physical findings in nine (4.3%) cases (Table 2).

**Table 2:** Mode of detection of first recurrence Total patients n=210

Mode of detection of first recurrence	Number of patients	Percentage
Symptoms	45	21.4%
Physical findings	9	4.3%
Raised CA-125	124	59%
Imaging	32	15.2%

Comparison of the site of recurrence with the mode of detection of first recurrence

While analysing the site of recurrence of 210 cases, 14 had biochemical recurrence without any symptoms, physical findings, or imaging findings. Hence, only 194 cases were analysed.

**Figure 1:**
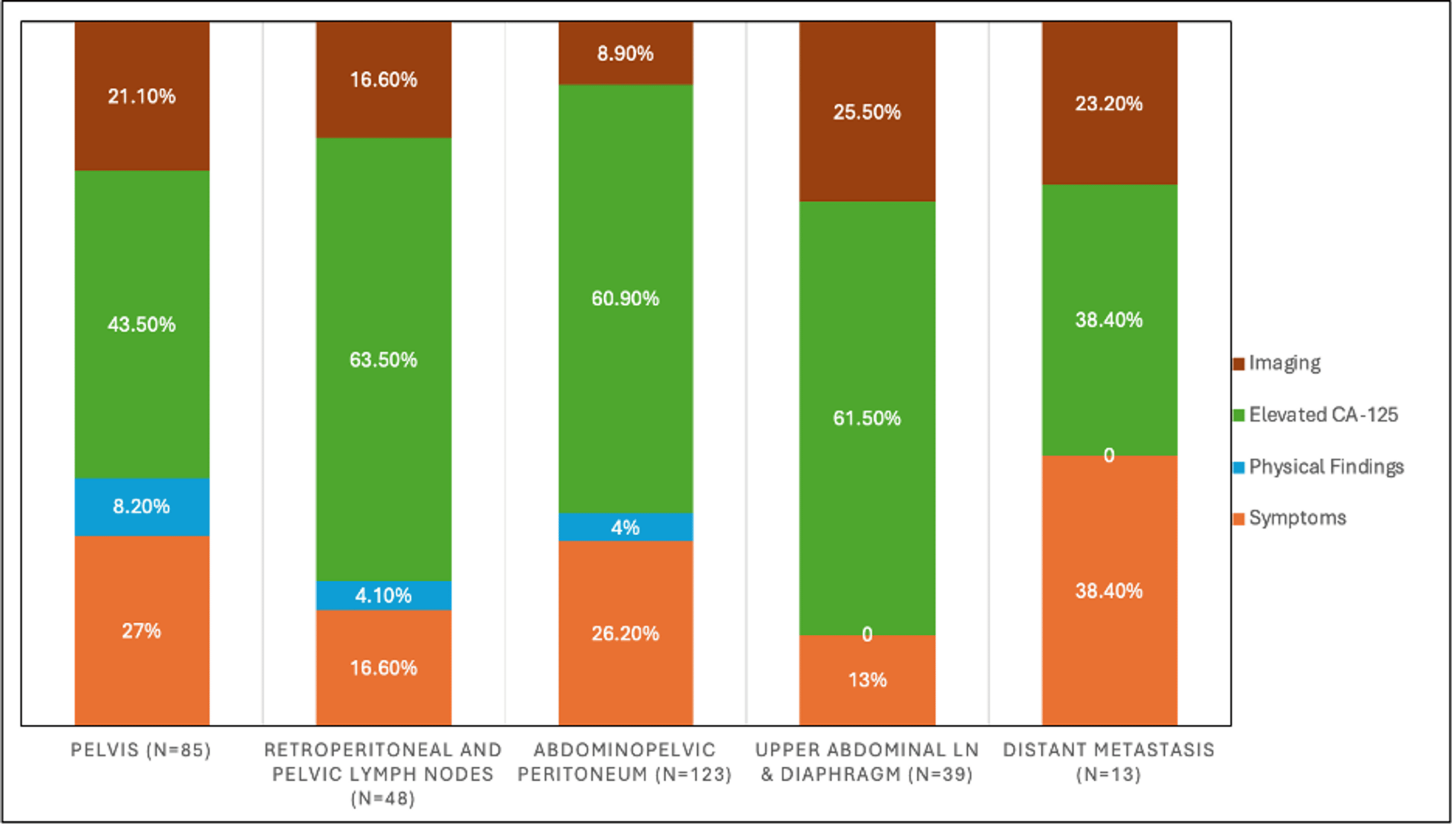
Distribution of the mode of detection of recurrence at each site Percentages are calculated using the total number of recurrences at each anatomical site as the denominator.

Figure 1 shows the distribution of the mode of detection of recurrence at each site. Elevated CA-125 levels were observed across most sites of recurrence. Among patients with pelvic recurrence (n = 85), 43.5% (n = 37) had raised CA-125 levels. CA-125 elevation was more frequent in retroperitoneal and pelvic lymph node recurrences (62.5%; n = 30/48) and abdominopelvic peritoneal recurrences (61.0%; n = 75/123). Similarly, 61.5% of patients with upper abdominal lymph node and diaphragmatic involvement (n = 24/39) demonstrated elevated CA-125 levels. In contrast, CA-125 elevation was less common in distant metastases, observed in 38.5% of cases (n = 5/13) (Figure 1).

However, when each site of recurrence was compared with the mode of detection, pelvic recurrences were most frequently detected by physical examination (7/9, 77%), followed by imaging (18/32, 56.2%), symptoms (23/44, 52.3%), and elevated CA-125 levels (37/112, 33%). In contrast, abdominopelvic peritoneal recurrences were most commonly identified through symptoms (32/44, 72.7%) and elevated CA-125 (75/109, 68.8%), while imaging detected a smaller proportion (11/32, 34.4%) (Table 3).

**Table 3:** Relation between the site of recurrence and the mode of detection of recurrence LN: Lymph node χ² and p value were calculated using Pearson's chi-square test. Percentages are calculated using the total number of cases detected by each mode of detection as the denominator.

Mode of detection of Recurrence	Pelvis			Retroperitoneal and pelvic LN			Abdominopelvic peritoneum			Upper abdomen LN & diaphragm			Distant metastasis		
	N (%)	P value	χ² value	N (%)	P value	χ² value	N (%)	P value	χ² value	N (%)	P value	χ² value	N (%)	P value	χ² value
Symptoms (n=44)	23 (52.3)	0.008	11.81	8 (18.2)	0.682	1.50	32 (72.2)	0.002	14.88	5 (11.6)	0.69	10.3	5 (11.4)	0.345	1.7
Physical findings (n=9)	7 (77.8)			2 (22.2)			5 (55.6)			-			-		
Elevated CA-125 (n=109)	37 (33.9)			30 (27.5)			75 (68.8)			24 (22)			5 (4.6)		
Imaging (n=32)	18 (56.2)			8 (25)			11 (34.4)			10 (31.3)			3 (9.4)		

Patient surveillance data

All the patients had a median of 6 (1-23) visits to the hospital before being detected with their first recurrence. The direct cost for the patient at every outpatient visit for surveillance was ₹1,500. This included the consultation fee and the cost of serum CA-125. Hence, the cost for a median of 6 (1-23) visits is approximately ₹9,000 (₹1,500-₹34,500).

The median distance travelled by the patients to reach the hospital was 70 km (4 km-1774 km).

The median follow-up period was 18 months (2-133 months) before the first recurrence was diagnosed. As per institutional practice, most of the patients were started on either second-line chemotherapy or metronomic therapy at the diagnosis of recurrence (Table 4).

**Table 4:** Patient surveillance data: travel metrics, visits, and costs

Metric	Value
Median distance travelled to reach a hospital	70 km (4-1174)
Median number of visits before being diagnosed with the first recurrence	6 visits (1-23)
Median surveillance time before being diagnosed with the first recurrence	18 months
Direct cost for outpatient visits during surveillance	₹9000 (₹1500-34,500)

**Figure 2:**
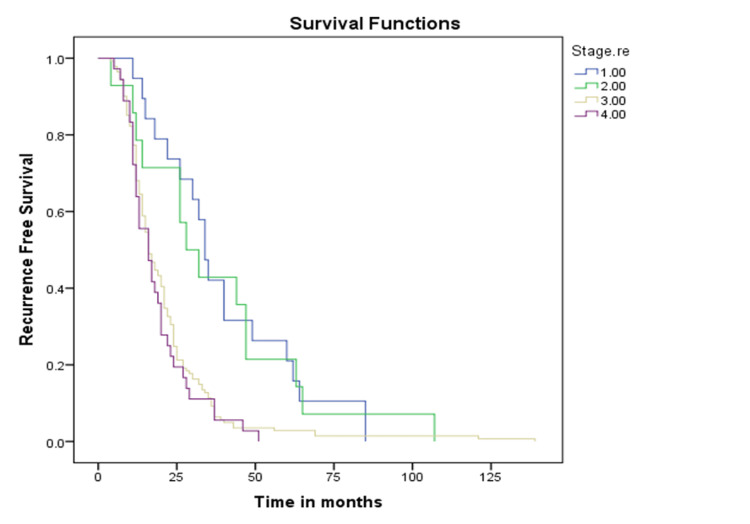
Kaplan-Meir curves showing the stages

Median DFS time was 18 months (15.7-20.2) with a three-year DFS of 14.8% and a five-year DFS of 5.4%. Median DFS time for stages I, II, III, and IV was 34, 28, 16, and 16 months, respectively (Figure 2).

## Discussion

This study on epithelial ovarian cancer recurrence reveals that raised CA-125 is the primary detection method of recurrence, accounting for 59% of cases, followed by symptoms at 21.4%. Imaging identifies recurrence first in 15.2% of cases, while physical findings are the initial mode of detection in only 4.3% of cases.

Recurrences in the pelvis and abdominopelvic peritoneum have a significant association with the mode of detection. Among the pelvic recurrences, physical examination proved to be the most effective (72.8%) when compared to elevated CA-125 levels (33%). Among abdominopelvic peritoneal recurrences, symptoms (72.7%) and elevated CA-125 (68.8%) were significantly higher when compared to imaging (34.4%).

Patients typically undergo a median of six hospital visits (range: 1-23), covering a median distance of 70 km (range: 4 km-1774 km) for each visit before recurrence diagnosis, with a median surveillance period of 18 months (range: 2-133 months) prior. The direct cost incurred by the patient for an outpatient visit during the surveillance period is approximately ₹9,000 (₹1,500-₹34,500). The study also reports a median DFS time of 18 months (range: 15.7-20.2), with three-year DFS and five-year DFS rates of 14.8% and 5.4%, respectively. Additionally, the median DFS times for stages I, II, III, and IV were 34, 28, 16, and 16 months, respectively.

The median disease survival for ovarian cancer patients ranges from 16 to 22 months [6,7] in the pre-PARP inhibitor era, which is similar to our study. However, with the routine use of targeted therapies in ovarian cancer treatment, the overall survival has improved [8]. These patients undergo asymptomatic surveillance despite the lack of strong evidence. In spite of scheduled follow-up protocols, there is no noticeable improvement in survival outcomes for patients who develop recurrent disease [9,10]. Given the limited maturity of the OS data in our cohort, we are unable to determine whether less frequent surveillance would influence survival outcomes. Additionally, 6.6% (14/210) of patients experienced biochemical recurrence, defined by elevated CA-125 according to predefined criteria, which may introduce lead-time bias in the interpretation of survival outcomes.

Studies done on surveillance in ovarian cancer patients in the past show that raised CA-125 is the most common method of detection of recurrence, which is similar to this study. A retrospective study among 147 patients by the Memorial Sloan Kettering Cancer Center (MSK) team revealed radiological imaging (55%), followed by raised tumour marker 33% as the most frequent means of detecting recurrence [5]. A retrospective study done by Janke et al. [11] among 272 patients questioned the utility of physical examination in routine surveillance in ovarian cancer, as only 24.8% of patients had abnormal physical findings, whereas raised CA-125 was noted in 89% of patients.

A retrospective Italian study showed that 80% of the patients were asymptomatic at the time of recurrence, and 34.5% were detected by a combination of raised CA-125 and imaging. Most had recurrences in the pelvis (30.6%), followed by the abdomen (24%), similar to our study [9].

Richardson et al. [10] conducted a retrospective cohort study to evaluate the role of asymptomatic screening in diagnosing and treating ovarian cancer recurrence. Even though 57% of recurrences were detected by raised CA-125, the method of detection did not impact survival or treatment outcomes.

The surveillance protocol followed worldwide varies and is based mainly on guidelines based on expert consensus. Armstrong et al. [12] evaluated the cost of CA-125 measurement, doctor visits, and imaging during ovarian cancer surveillance for a cohort of 60 patients. Elevated CA-125 levels identified a substantial 57% of cancer recurrences, with 95% detected through either CA-125 monitoring or hospital visits and only 5% detected by imaging alone. The surveillance cost nearly doubled when imaging was included in the surveillance protocol. The surveillance cost for this population for two years was $32,500,000 using NCCN guidelines and $58,000,000 if one CT scan was obtained.

In India and most developing countries, healthcare is private, and the government health schemes and insurance policies are insufficient to cover all costs. Hence, the financial burden lies on the patient and family. Cancer-related care faces high out-of-pocket expenses, driving the patient and family into a severe financial strain, which increases the catastrophic health expenditure [13].

A systematic review [14] on the financial burden of non-communicable diseases among low- to middle-income countries showed that the average cost per year for a cancer patient was $3,303.81. The average cost of outpatient consultation and inpatient hospitalisation in cancer centres in India is estimated to be ₹8,053 (US$101) and ₹39,085 (US$492), respectively, with diagnostics contributing to 38% of the costs [15].

There are other studies done in India, which show that the average out-of-pocket expenditure for cancer treatment ranges from ₹57,000-₹84,000, which is met by borrowing money and the sale of assets [16-18]. Higher costs for surveillance post-primary treatment of ovarian cancer are seen even in developed countries. Different cost-effective surveillance strategies were studied in the USA, and the most cost-effective and the highest detection of recurrence, strategy cost $9.2 million annually, which included office visits biannually, biannual CA-125 levels, and annual asymptomatic imaging [19].

At our tertiary care multispecialty teaching hospital, where patients benefit from subsidised rates, the direct costs for an outpatient consultation combined with a CA-125 blood test amount to approximately ₹9,000, ranging from ₹1,500 to ₹34,500 depending on the median number of visits. The cost escalates further with the addition of a CT scan of the abdomen and pelvis, priced at ₹12,000. Moreover, out-of-pocket expenses, such as transport and loss of daily wages, add to the financial burden, making surveillance a significant economic challenge for many patients.

A 2018 Cochrane [20] review concluded that routine surveillance with CA-125 and treating asymptomatic relapses does not improve survival rates compared to treating symptomatic relapses. However, most patients seek reassurance that their cancer has not recurred and value the confirmation from their physician that there are no signs of disease [21]. Therefore, a more balanced approach to surveillance is necessary to support patients' psychological well-being without compromising oncological outcomes. In a systematic review by Dahl et al. [22] on quality of life and follow-up preferences after diagnosis of gynaecological cancer, the most significant concern was a recurrence. The patients presumed that surveillance programs were designed to address recurrence. However, when they were informed about the lack of evidence on surveillance, most of them preferred “point of need care.”

This study faced limitations inherent to retrospective cohorts. The study was conducted at a tertiary referral centre, and some patients sought treatment closer to their homes, leading to incomplete medical records and exclusion from the study, which increased the selection bias. Despite these limitations, our study stands among the few that focus attention on ovarian cancer surveillance. Moreover, it sheds light on the financial and psychosocial aspects affecting patients, contributing valuable insights to the field.

## Conclusions

This study suggests that the first recurrence of epithelial ovarian cancer is most often detected through elevated CA-125 levels and patient-reported symptoms, prompting a re-evaluation of the utility of routine hospital visits for physical examination. It underscores the substantial financial and emotional burden associated with frequent follow-up, particularly the number of visits and long travel distances faced by patients and their families in low-resource settings. In this context, telemedicine-based follow-up and greater involvement of local general physicians may offer pragmatic alternatives to reduce this burden while maintaining timely detection. Prospective studies are needed to develop cost-effective, patient-centred surveillance protocols that align with healthcare system capacities without compromising clinical outcomes.
